# Supplementary material for: eHealth literacy and its associated factors in Ethiopia: Systematic review and meta-analysis
Source: PLoS One. 2023 Mar 3;18(3):e0282195. doi: 10.1371/journal.pone.0282195 (PMC9983868; doi:10.1371/journal.pone.0282195)
Supplement: Attachment — Submitted filename: Rebuttal letter.docx [file pone.0282195.s004.docx]

**Dear Editors of PLOS ONE :**

It has been recalled that we the authors of the manuscript entitled “**eHealth Literacy and its associated factors in Ethiopia: Systematic Review and Meta-Analysis**” submitted our manuscript for publication in your journal and received reviewer comments for the betterment of the manuscript before its publication. In line with this, all authors are very happy with the constructive and valuable comments given by reviewers. Accordingly, we have considered all the comments and provided a point-by-point response and explanations for all the questions raised. Finally, we have submitted all the required documents in their revised form. We hope that we have addressed all the questions and if you have any points for further clarity, let us know.

All the authors would like to thank the editorial team and reviewers

**Editor(s)’ comments to the authors**

**Comment1:** A rebuttal letter that responds to each point raised by the academic editor and reviewer(s). You should upload this letter as a separate file labeled 'Response to Reviewers'.

**Answer:** Thanks dear editor for your nice comments and suggestions. We, the authors
of this study, have attached the necessary files and a detailed rebuttal letter according to
your suggestion and the journal format.

**Comment2:** A marked-up copy of your manuscript that highlights changes made to the original version. You should upload this as a separate file labeled 'Revised Manuscript with Track Changes.

**Answer:** Thank you, the track changes and cleaned document have been prepared and labeled as revised manuscript and attached

**Comment 3:** An unmarked version of your revised paper without tracked changes. You should upload this as a separate file labeled 'Manuscript'.

**Answer:** thank you very much, dear editor, unmarked version of the revised manuscript was prepared without track change labeled as the manuscript was uploaded.

**Journal Requirements:**

**Comments:** Please review your reference list to ensure that it is complete and correct. If you have cited papers that have been retracted, please include the rationale for doing so in the manuscript text, or remove these references and replace them with relevant current references. Any changes to the reference list should be mentioned in the rebuttal letter that accompanies your revised manuscript. If you need to cite a retracted article, indicate the article’s retracted status in the References list and also include a citation and full reference for the retraction notice.

**Answer:** thank you dear editor we authors of this study agreed with your comments and suggestions. We tried to review the whole references cited in the manuscript are complete and correct. we ensured that all references are correct and complete no retracted papers are cited in our manuscript.

**Reviewer 1 comment to the author**

**Comment#reviwer1:**  Paper must be improved in all sections. It is not clear your statistical achievements of the paper. If any, it is not too evident to the reader. In your abstract, it was written the numerical results. A Journal paper requires transparently your exact methodology about how you got such statistical results. Finally, it is not common that research of such importance as ehealth systems in Ethiopia have only 47 words as a conclusion.

**Answer:** thank you very much for your valuable comments ad suggestion for the improvement of our manuscript, we authors are happy to accept and agreed with the comments. Accordingly, we revised all sections as recommended in the main revised manuscript. The abstract section is written clearly and in an understandable manner. The method, result, and discussion section are refined again in the revised manuscript.

**Comment Reviewer #2:** The heterogeneity test is the homogeneity test of statistics, which aims to check whether there is heterogeneity in the results of independent studies. If there is no or low heterogeneity among the studies (P＜0.1, heterogeneity level＞50%), the fixed effect model is used for data consolidation analysis; If there is heterogeneity (P ＜ 0.1, heterogeneity level ＞ 50%), but the combined data still have clinical significance, the random effect model is used for combined analysis

**Answer#reviwer2:** thank you very much dear reviewer for your insightful, fruitful, and constructive comments to improve our manuscript document development. We authors of this study gladly agreed and accepted all your concerns. Hence the result of this meta-analysis showed that there is no heterogeneity between individual articles, we employed fixed effect model analysis. However, in the submitted paper we say that the random effect model was used, it was wrongly written and corrected in the revised manuscript.

**Comment Reviewer #3:** Title of the manuscript: eHealth Literacy and its associated factors in Ethiopia: Systematic review and meta-analysis Thank you for the opportunity to review a timely and interesting manuscript. I look forward to seeing the manuscript in print, and the comments below are meant to strengthen the manuscript towards this end. The study aimed to generate the pooled magnitude of e-health literacy and its predictors among adults in Ethiopia. Much as I appreciate the enormous amount of hard work put in by the author(s) and the potential contributions of their study, I am afraid that reworking will be required before the manuscript can be considered for publication. I will outline some major issues below:

**Answer Reviewer #3::** thank you very much, dear reviewer, for your appreciation of our work and for giving constructive comments on our paper's advancement. We authors of this study gladly accepted and agreed with your comments. Accordingly, refined all sections in the revised manuscript.

**Comment Reviewer #3:** Given the large and diverse potential audiences for this work, the author(s) might want to be more explicit about the exclusion of “Gray and unpublished artifacts were excluded.”

**Answer Reviewer #3:**  thank you so much for your valuable contribution to improving this manuscript document, we are happy to accept your interesting comment. Since gray and unpublished papers are not passed through the previewed process the information gained from them is not helpful for decision-making and policy development. the uncertainty of the status of this information generated from such articles.

The findings mentioned some major findings, but it is not immediately evident what new findings the study has produced (or what is unique about the study?). For example:

**Comment Reviewer #3** (a): The statement “However it is significantly lower than studies conducted in (12-16). This might be due to differences in socioeconomic status, digital technology penetration, educational system, and study participants.” (So, what about it? What can be inferred from this finding or possibly the differences mentioned? Signify? Implied?)

**Answer Reviewer #3:** thank you dear reviewer for your interesting comments and suggestion forwarded to our manuscript advancement. Accordingly, we tried to show the inference of the finding in a more compressive way in the revised manuscript

**Comment Reviewer #3** (b)The statement “The same findings were obtained suggesting a strong correlation between the perceived usefulness of the Internet to make health decisions and the level of eHealth literacy (17-19).this is due to knowing about internet users will have an impact on increasing eHealth literacy of individuals. (Please re-write for clarity)

**Answer Reviewer #3:** thank you so much for the nice comments, we authors of this study gladly accept and agree with this comment. Accordingly, we wrote the above reason in a clear way that the reader can understand in the revised manuscript.

**Comment Reviewer #3** (c) The statement “This finding is consistent with studies done in (20-23).this could be having internet access will enforce the individual to search about digital technologies applications on health.” (Apart from consistent findings with other studies, what can you imply or interpret from it? and How?)

**Answer Reviewer #3:** thank you very much, dear reviewer, we are happy to accept your comments on the improvement of our manuscript. accordingly, we tried to show interpreting reasons rather than listing them in the revised manuscript.

**Comment Reviewer #3:** Much of this section is merely descriptive with some interpretation. Critical interpretation and connection are missing. The author(s) might want to be more explicit about the significant contribution that the study made to the advancement of knowledge and understanding in the chosen area of study. Overall, I believe the discussion needs to/can be strengthened to highlight more explicitly the link between the relevant factors of e-health literacy.

**Answer Reviewer #3:** thank you, we author of this study accepted and agreed on your recommended comments to our manuscript suite for publication. Accordgly, we authors refined more this manuscript by showing the significant contribution to generating knowledge on the area. The discussion part strengthens and tries to show factors associated with eHealth literacy level.

Limitation:
**Comment Reviewer #3:** There should be a critical reflection on the limitations of the study (systematic review and meta-analysis) before the recommendations. In the current form, the limitation lacks criticality and interpretation of the impact.

**Answer Reviewer #3:** thank you so much for your insightful comments on this manuscript, we authors of this study agreed on that comment. Accordingly, we showed the limitation of this systematic review and meta-analysis study in a more comprehensive manner to show clear evidence in the revised manuscript.

Conclusion:
**Comment Reviewer #3**. A conclusion is presented in the manuscript but is insufficient. The question of ‘where do we go from here?’ is not presented to the reader(s). The author(s) may consider succinctly informing the reader(s) how and why it is that what has been presented in this manuscript is significant for policy, practice, or further research or action. The conclusion section should/must also address the implications.

**Answer Reviewer #3:** thank you dear reviewer for your interesting comments forwarded to our manuscript development. So, we tried to show the conclusion section of the manuscript more about the implication of the finding and future directions in the revised manuscript
