# Supplementary material for: eHealth literacy and its associated factors in Ethiopia: Systematic review and meta-analysis
Source: PLoS One. 2023 Mar 3;18(3):e0282195. doi: 10.1371/journal.pone.0282195 (PMC9983868; doi:10.1371/journal.pone.0282195)
Supplement: Attachment — Submitted filename: Reviewer comments for PLOS ONE.docx [file pone.0282195.s003.docx]

**Reviewer comments for PLOS ONE_PONE-D-22-21015**

**Title of the manuscript: eHealth Literacy and its associated factors in Ethiopia: Systematic review and meta-analysis**

Thank you for the opportunity to review a timely and interesting manuscript. I look forward to seeing the manuscript in print, and the comments below are meant to strengthen the manuscript towards this end.

The study aimed to generate the pooled magnitude of e-health literacy and its predictors among adults in Ethiopia. Much as I appreciate the enormous amount of hard work put in by the author(s) and the potential contributions of their study, I am afraid that reworking will be required before the manuscript can be considered for publication. I will outline some major issues below:

**Study selection criteria:**

Given the large and diverse potential audiences for this work, the author(s) might want to be more explicit about the exclusion of “Gray and unpublished artifacts were excluded.”

**Discussion:**

The findings mentioned some major findings, but it is not immediately evident what new findings the study has produced (or what is unique about the study?). For example:

(a) The statement “However it is significantly lower than studies conducted in (12-16). This might be due to differences in socioeconomic status, digital technology penetration, educational system, and study participants.” (So, what about it? What can be inferred from this finding or possibly the differences mentioned? Signify? Implied?)

(b) The statement “The same findings were obtained suggesting a strong correlation between the perceived usefulness of the Internet to make health decisions and the level of eHealth literacy (17-19).this is due to knowing about internet users will have an impact on increasing eHealth literacy of individuals. (Please re-write for clarity)

(c) The statement “This finding is consistent with studies done in (20-23).this could be having internet access will enforce the individual to search about digital technologies applications on health.” (Apart from consistent findings with other studies, what can you imply or interpret from it? and How?)

Much of this section is merely descriptive with some interpretation. Critical interpretation and connection are missing. The author(s) might want to be more explicit about the significant contribution that the study made to the advancement of knowledge and understanding in the chosen area of study. Overall, I believe the discussion needs to/can be strengthened to highlight more explicitly the link between the relevant factors of e-health literacy.

**Limitation:**

6. There should be a critical reflection on the limitations of the study (systematic review and meta-analysis) before the recommendations. In the current form, the limitation lacks criticality and interpretation of the impact.

**Conclusion:**

7. A conclusion is presented in the manuscript but is insufficient. The question of ‘where do we go from here?’ is not presented to the reader(s). The author(s) may consider succinctly informing the reader(s) how and why it is that what has been presented in this manuscript is significant for policy, practice, or further research or action. The conclusion section should/must also address the implications.
